# Holistic Recollection via Pattern Completion Involves Hippocampal Subfield CA3

**DOI:** 10.1523/JNEUROSCI.0722-19.2019

**Published:** 2019-10-09

**Authors:** Xenia Grande, David Berron, Aidan J. Horner, James A. Bisby, Emrah Düzel, Neil Burgess

**Affiliations:** ^1^German Center for Neurodegenerative Diseases, 39120 Magdeburg, Germany,; ^2^Institute of Cognitive Neurology and Dementia Research, Otto-von-Guericke University Magdeburg, 39120 Magdeburg, Germany,; ^3^Institute of Cognitive Neuroscience, University College London, London WC1N 3AZ, United Kingdom,; ^4^UCL Queen Square Institute of Neurology, University College London, London WC1N 3BG, United Kingdom,; ^5^Wellcome Trust Centre for Human Neuroimaging, University College London, London WC1N 3AR, United Kingdom,; ^6^Clinical Memory Research Unit, Department of Clinical Sciences Malmö, Lund University, 223 62 Lund, Sweden,; ^7^Department of Psychology, University of York YO10 5DD, United Kingdom, and; ^8^York Biomedical Research Institute, University of York YO10 5DD, United Kingdom

**Keywords:** 7 T fMRI, hippocampal subfields, medial temporal lobe, memory, pattern completion, recollection

## Abstract

Episodic memories typically comprise multiple elements. A defining characteristic of episodic retrieval is holistic recollection, i.e., comprehensive recall of the elements a memorized event encompasses. A recent study implicated activity in the human hippocampus with holistic recollection of multi-element events based on cues (Horner et al., 2015). Here, we obtained ultra-high resolution functional neuroimaging data at 7 tesla in 30 younger adults (12 female) using the same paradigm. In accordance with anatomically inspired computational models and animal research, we found that metabolic activity in hippocampal subfield CA3 (but less pronounced in dentate gyrus) correlated with this form of mnemonic pattern completion across participants. Our study provides the first evidence in humans for a strong involvement of hippocampal subfield CA3 in holistic recollection via pattern completion.

**SIGNIFICANCE STATEMENT** Memories of daily events usually involve multiple elements, although a single element can be sufficient to prompt recollection of the whole event. Such holistic recollection is thought to require reactivation of brain activity representing the full event from one event element (“pattern completion”). Computational and animal models suggest that mnemonic pattern completion is accomplished in a specific subregion of the hippocampus called CA3, but empirical evidence in humans was lacking. Here, we leverage the ultra-high resolution of 7 tesla neuroimaging to provide first evidence for a strong involvement of the human CA3 in holistic recollection of multi-element events via pattern completion.

## Introduction

Episodic memories bind multiple elements into a single representation. Recollection may be triggered by any one of these elements. Asked, for example, about whether we had been to a certain restaurant before, we may recall meeting a friend there lately. Remarkably, the “restaurant” cue may even initiate *holistic* recollection: another guest's dog or the piano in the restaurant may come to our mind. Holistic recollection thus refers to comprehensive recall of the elements an event encompasses, even though incidental to the current situation (Tulving, 1983).

Successful pattern completion is considered a prerequisite for such holistic recollection. The cue information needs to be completed toward the full event to produce comprehensive recall (Marr, 1971; Treves and Rolls, 1994; McClelland et al., 1995). A corresponding feature of recollective experiences is the reinstatement of the encoding-related cortical activity (Staresina et al., 2012, 2013; Bosch et al., 2014; Gordon et al., 2014; Liang and Preston, 2017). Recently, it has been shown that cortical reinstatement of incidentally recalled event elements is related to functional activity in the hippocampus (Horner et al., 2015). However, the spatial resolution was not sufficient to dissect the specific involvement of hippocampal subfields.

Anatomically inspired computational and theoretical models attribute different information processing mechanisms to different hippocampal subfields. Unique recurrent collaterals in subfield CA3 provide an effective condition for the implementation of pattern completion (Marr, 1971; Treves and Rolls, 1991). Consequently, computational models suggest subfield CA3 to guide the incidental recall of additional event elements based on pattern completion (Treves and Rolls, 1994; McClelland et al., 1995).

Empirical support for the functional role of CA3 in pattern completion mainly originates from animal research (Nakazawa et al., 2002; Lee and Kesner, 2004; Vazdarjanova and Guzowski, 2004; Gold and Kesner, 2005; Fellini et al., 2009; Neunuebel and Knierim, 2014). Until recently the resolution of human functional magnetic resonance imaging (fMRI) did not allow to separate subfield CA3 from dentate gyrus (DG). Therefore, most fMRI studies indiscriminately attribute pattern completion to human subfield CA3/DG (Chen et al., 2011; Dudukovic et al., 2011; Schapiro et al., 2012; Hindy et al., 2016). Solely Bonnici et al. (2012) and Chadwick et al. (2014) demonstrated a generalization function selectively in CA3. Evidence for explicit functional engagement of (the human) CA3 in holistic recollection and thus mnemonic pattern completion is still pending.

Here, we aimed to provide first empirical evidence at the hippocampal subfield level for the functional underpinnings of holistic recollection via pattern completion in humans using fMRI data with ultra-high resolution at 7 tesla. We used the same task as Horner et al. (2015) during which multi-element events were learned as overlapping pairs of associations between elements (places, people, and objects), and subsequently retrieved as paired associations. This task allowed us to assess holistic recollection both behaviorally and in terms of neural activity. That is, we calculated the statistical dependency in performance of retrieving one association from an event on retrieving another association from the same event. We also measured the extent of incidental retrieval of event elements that were neither the cue nor target of retrieval in terms of regional activity during retrieval corresponding to the nontarget element category (e.g., place, people, or object). Fully overlapping associations (closed-loops), which appear to create coherent events with holistic recollection, were compared with partially overlapping associations (open-loops); for details, see Horner et al. (2015). We hypothesized that cortical reinstatement of incidental elements during holistic recollection would be associated with activity in hippocampal subfield CA3 but not DG.

## Materials and Methods

### Participants

In total, 30 participants [12 female, mean (SD) age: 27 (4)] were recruited from the campus of Otto-von-Guericke University Magdeburg and the Leibniz Institute for Neurobiology Magdeburg. All participants reported to be right-handed and without any neurological or psychiatric illness. If necessary, vision was corrected to normal. Minimum educational level of all participants was the German Abitur (A-level). The participants received an allowance of 30 €. The study was approved by the local Ethics Committee of the Otto-von-Guericke University Magdeburg.

### Materials and procedure

Regarding materials and procedure we follow Horner et al.'s (2015) setup closely. In the following sections, the main features of the design are outlined and adjustments that were necessary are specified.

#### Materials

Stimuli consisted of written words that belonged to four categories: locations (e.g., kitchen), objects (e.g., hammer), animals (e.g., mouse), and famous people (e.g., Obama). The words were taken from Horner et al. (2015) and translated into German. To assure a similar level of familiarity within our German sample, several people-stimuli were changed based on preceding behavioral pilot results. In total, 36 events were created by associating one example out of each category with another. Initially, four event sets were built and randomized across participants. For each participant, 18 events were assigned randomly to consist of four categories (location, object, people, animal). These events will be referred to as open-loop structure events in the following. The remaining 18 events consisted of three categories. Within these closed-loop structure events, nine events were randomly selected to encompass the categories location–object–people and nine events to encompass the categories location–animal–people.

Words were presented in white font on a black background to the center of a screen (font size = 30) and via a mirror mounted on the head coil, participants could watch the projected screen with a visual angle of ± 3° × ± 2°.

#### Task procedure

Before the scanning session, participants received task instructions. The task was described as an associative learning paradigm. They were told to imagine each displayed associative word pair together in one scene as vividly as possible. Importantly, the underlying associative event structure of the stimuli was not revealed and remained implicit.

**Figure 1.**
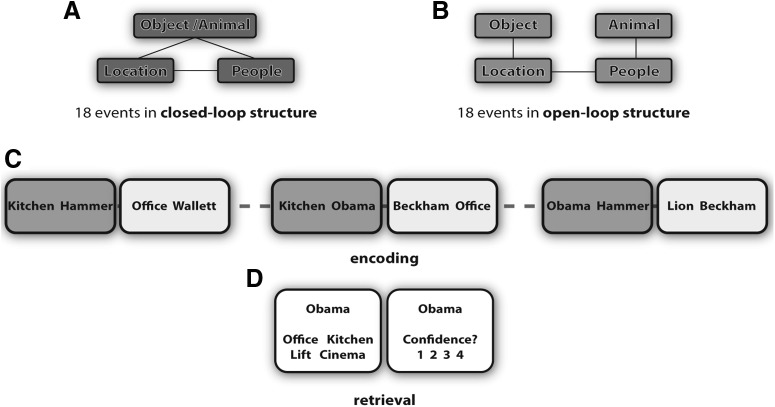
Multi-element event paradigm (Horner et al., 2015). Participants learned 36 events that consisted of multiple elements, with each element belonging to the location, people, or object/animal category. All events followed either a closed-loop structure (***A***) or an open-loop structure (***B***). ***C***, At encoding, events were learned in three blocks in a pairwise associative manner, one associative pair at each block. ***D***, At retrieval, all three pairwise associations within each event were tested bidirectionally. The four-alternative forced choice recognition trial was followed by a confidence rating.

During the scanned encoding phase, participants learned the 36 events in a pairwise associative manner. The encoding phase consisted of 3 blocks with 36 trials each, adding up to a total of 108 encoding trials. In each block, one associative pair of each event was presented for 6 s (e.g., kitchen–hammer out of the event kitchen–hammer–Obama–dog; Fig. 1*C*). Following that procedure, one element within an event overlapped between the first and the second encoding block. At the third block, some events remained as an associative chain and followed an “open-loop” event structure (Fig. 1*B*). Thus, in the last encoding block, the third associative pair from these events overlapped again with one element from previously encoded associates of the respective event (AB–BC–CD). In contrast, “closed-loop” events were structured such that at the last encoding block both elements of the currently encoded associate overlapped with previously encoded elements from the respective event (AB–BC–CA; Fig. 1*A*).

The specific category pairing at each block was randomized. However, the third encoding block was restricted to a location–object/animal or a people–object/animal category pair. Further details about the randomization procedure can be found in the study by Horner et al. (2015). No responses were required by the participants. The interstimulus interval was 1500 ms and each encoding trial was initiated with a fixation cross of 500 ms.

The scanned retrieval phase followed encoding immediately. Here, each pairwise association within an event was tested. This yielded six retrieval trials per event and 215 retrieval trials in total. The six retrieval trials were distributed over six blocks. During each block one associative pair from each event was tested, each pair bidirectionally. On each trial, participants were cued with one element from an event and instructed to retrieve an associated element by means of a four-alternative forced-choice recognition procedure (Fig. 1*D*). The displayed lures belonged to the same category as the target but were taken from other encoded events. Cue and response options were presented until a response was made but with a maximum of 6 s. For further details on the randomization procedure at retrieval, see Horner et al. (2015). Each retrieval trial was followed by a 1–4 confidence rating for 6 s. The interstimulus interval was 1500 ms and each retrieval trial was initiated with a 500 ms fixation cross.

A debriefing phase of ∼30 min immediately followed the scanning session. More details regarding the administered questions can be found in the study by Horner et al. (2015).

#### Scanning procedure

The scanning was performed with a 7 tesla MRI Siemens machine. A 32-channel head coil was used. Participants received earplugs and ear defenders to protect against noise. Before functional data acquisition, structural images were acquired. First, a whole-brain T1-weighted volume was obtained (TR = 2300 ms; TE = 2.73 ms; flip angle = 5°; resolution = 0.8 mm isotropic; matrix size = 320 × 320). Second, a partial high-resolution T2-weighted volume was acquired with an orientation aligned orthogonally to the hippocampal main axis (TR = 8000 ms; TE = 76 ms; slice thickness = 1 mm with 1.1 mm slice spacing; in-plane resolution = 0.4375 mm × 0.4375 mm; 55 coronal slices; FOV = 256 mm × 256 mm; matrix size = 512 × 512).

Succeeding the structural data acquisition, two runs of functional data were obtained. Both runs consisted of T2*-weighted echoplanar slices (EPI), oriented in parallel to the hippocampal long axis (28 axial slices; TR = 2000 ms; TE = 22 ms; matrix size 1536 × 1536; FOV = 256 mm × 256 mm; resolution = 0.8 mm, odd–even interleaved slice acquisition). First, functional data regarding the encoding phase were obtained (440 volumes). Second, the functional data regarding the retrieval phase were obtained (∼700 volumes, depending on response times). Responses were recorded using a scanner-compatible four-choice button box. The complete scanning procedure took ∼80 min.

The functional data were distortion-corrected by means of a point spread function (Zaitsev et al., 2004) and online motion corrected during image reconstruction.

### Behavioral data analyses

The overall accuracy per participant was calculated as the percentage of correct retrieval trials. Note that there are 6 retrieval trials for each of the 36 events. We calculated accuracy separately for closed- and open-loop events. With a paired samples *t* test, we tested for significant differences in performance between loop conditions (closed- vs open-loop events). We also evaluated the amount of retrieval dependency among the elements within an event, separately for closed- and open-loop events. This measure reflects the likelihood that an element is successfully retrieved, given successful retrieval of the other elements that belong to the same event. The dependency measures were calculated by means of participant-specific contingency tables. In total, six contingency tables were created per participant, one for each category [location (A), people (B), object (C)] being either cue or target. The cue-based tables reflect the retrieval dependency of two elements from the same event across separate retrieval trials, given the trials used the same cue element from the respective event (AbAc). The target-based tables reflect the retrieval dependency of the same target element across separate retrieval trials, given the trials used different cue elements belonging to the same event (BaCa). Each table's cells contain the retrieval performance across events for the respective condition. The dependency measure based on observed data are defined as the proportion of events for which both overlapping associations related to a common element (either being cue or target) are retrieved successfully or unsuccessfully.

To assess the dependency measures from the data, we compared them with both a model that assumes full retrieval dependency, and a model that assumes full retrieval independency among all elements of an event. The expected dependency based on the independent model was estimated by multiplying the probabilities of separately retrieving either of the two items of an event within the contingency tables. The dependent model is based on the independent model but estimates the expected dependency by accounting for the level of guessing and inserting an “episodic factor”. This episodic factor weights the performance for a certain event by a factor that captures the difference between the respective event's performance across separate retrieval trials versus general performance across all events. Note, that the measure of observed dependency scales with accuracy. Therefore, only comparisons between observed dependency measures and model-based expected dependency values are informative. Comparisons between dependency measures were made using paired-sample *t* tests for both event structure conditions (open-loop and closed-loop), separately. For further details on the calculation of dependency measures based on the data and based on the two models, see Horner et al. (2015) and Horner and Burgess (2013).

To gain an impression of dependency differences that might be masked due to high accuracy levels in both loop conditions (88.55% and 86.27% for closed- and open-loop, respectively), the confidence level was taken into account. Dependency measures were evaluated in the above-described manner. However, instead of calculating dependency measures based on contingency tables that refer to correct versus incorrect retrieval, now the contingency tables were refined to reflect high confidence (Scores 3 or 4) versus low confidence (Scores 1 or 2) or incorrect retrieval. Statistical comparisons between dependency scores in different event loop conditions were made with paired-sample *t* test. As indicated above, these comparisons involve the differences in observed dependency and expected dependency based on the independent model in respective conditions.

### Functional data analyses

#### Preprocessing

All preprocessing steps were performed with SPM12 (Statistical Parametric Mapping v12, Wellcome Trust Centre for Neuroimaging, University College London; RRID:SCR_007037; Penny et al., 2011). The raw functional data were distortion- and motion-corrected already (see Materials and procedure: Scanning procedure). First, the raw data were converted from DICOM into NifTi format. Second, slice timing correction was applied and the data were smoothed with a full-width half-maximum Gaussian kernel of 2 × 2 × 2 mm. The size of the kernel was chosen based on previous reports to preserve high specificity but increase sensitivity at the same time (Maass et al., 2015; Berron et al., 2016).

Outliers based on motion (threshold 2 mm) or global signal (threshold 9.0) were detected by the ARTifact detection Tools (ART) software package (RRID:SCR_005994; Mozes and Whitfield-Gabrieli, 2011). The fully preprocessed data were used for outlier detection. The procedure resulted in a vector for each participant that indicated outlier scans. They were entered as separate regressors into all univariate analyses (see Functional analyses in detail).

#### Structural template calculation (T1 weighted)

To calculate and visualize functional analyses results on group level, a sample-specific template was created for the T1-weighted structural volumes. This assures optimal alignment of the functional data across participant (Avants et al., 2011). We used the nonlinear diffeomorphic mapping procedure called “buildtemplateparallel.sh” provided by Advanced Normalization Tools (ANTS) to construct a T1-template based on the 30 whole-brain T1-weighted volumes obtained from all participants (RRID:SCR_004757; Avants et al., 2010).

#### Hippocampal segmentation

The current study aimed to examine specific functional activity patterns in the hippocampus (HC). Thus, we restricted several functional analyses (indicated below) to hippocampal regions-of-interest (ROIs). Using ITK-SNAP (RRID:SCR_002010; Yushkevich et al., 2006) we manually segmented the bilateral hippocampus in all 30 participants on their specific T2-weighted structural volume. Therein we followed the segmentation protocol by Berron et al. (2017). This yielded participant-specific masks for HC subfields CA1, CA2, CA3, subiculum, and DG, one for each hemisphere.

**Figure 2.**
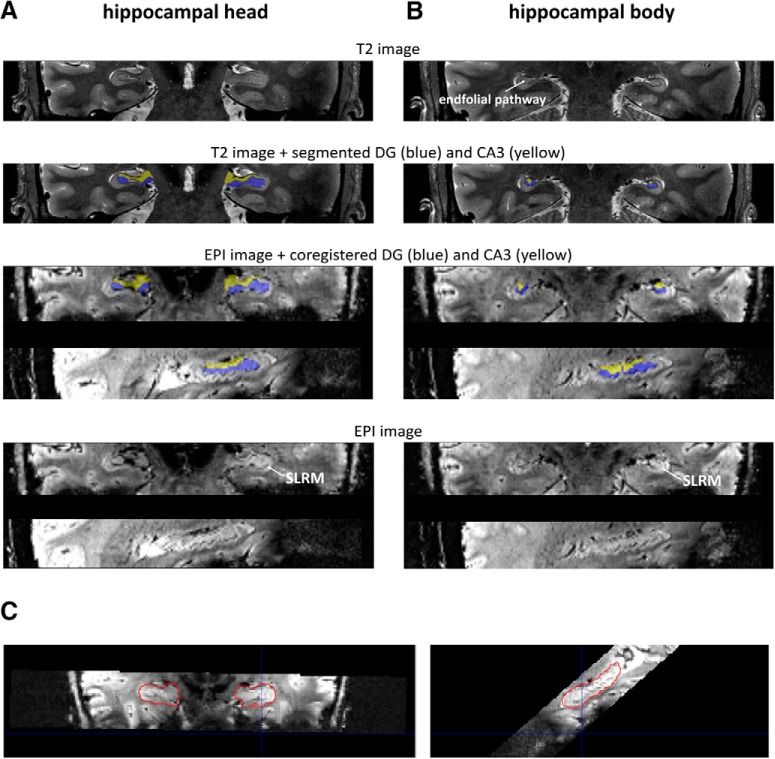
Example segmentation of hippocampal subfield DG (blue) and CA3 (yellow) and coregistration from T2 to EPI space. The displayed images correspond to one participant. Manual segmentation was performed on individual T2 images (Berron et al., 2017). Segmented masks were then coregistered to the individual EPI space. Here, the coregistered masks are displayed on the participant's mean EPI; the lowest panel corresponds to the respective mean EPI. Crucial hippocampal features for the segmentation (stratum radiatum/stratum lacunosum-moleculare (SLRM) and the endfolial pathway on T2 images) are indicated. Two corresponding slices in T2 and EPI space are shown from the hippocampal head (***A***) and the hippocampal body (***B***). A sagittal view on the coregistration between an individual EPI and the segmented hippocampal mask in T2 space (red outline) is presented in (***C***). DG, dentate gyrus; CA3, cornu ammonis 3; EPI, echo-planar image.

To use these masks as anatomical regions of interests in the functional analyses, each participant-specific T2-weighted HC subfield mask was coregistered to the participant's EPI-space and resampled to the EPI-resolution. This was accomplished in two steps. First, SPM12 was used to co-register and resample the T2-weighted HC subfields masks to the individual T1 space by applying “spm_coreg” (Penny et al., 2011). Second, these masks where coregistered from the individual T1 space to the EPI space using FSL FLIRT (RRID:SCR_002823; Greve and Fischl, 2009; Jenkinson and Smith, 2001; Jenkinson et al., 2002). See Figure 2 for an example segmentation and coregistration from T2 to EPI space.

All masks were divided in an anterior and a posterior part. To that end, the main hippocampal extension in each hemisphere was defined for each individual by taking the outer parts of the *z* dimension. All hippocampal subfields of that participant within that hemisphere were split in two at the border identified by half the length of the total hippocampus in *z* direction.

#### General functional analyses approach

All functional analyses were performed with SPM12 (Penny et al., 2011) on single participant and group levels.

##### Functional analysis at the participant level.

At the first level, a general linear model was fit to each participant's functional data in native space. Therefore, the underlying neural data were modeled by a boxcar function at stimulus onset for each condition-of-interest (dependent on the respective analysis). The resulting neural model was convolved by a canonical hemodynamic response function to predict the functional data. In addition to the regressors predicting the functional data related to each condition of interest, each general linear model also included one intercept regressor and six motion correction parameters as regressor of no interest. The motion-correction parameters were added to capture variability related to task-correlated motion and reduce the amount of false-positive activity in task conditions (Johnstone et al., 2006). If applicable, a regressor of no interest was added to capture variance in the functional data related to the outlier scans. Each general linear model was fit to the acquired functional data to obtain parameter estimates for each condition of interest. To examine differences in BOLD activity related to the conditions of interest, contrast maps were calculated for each participant in native space (specific contrasts dependent on respective analysis).

##### Normalization.

To be able to assess consistent contrast effects at group level, we normalized each participant's contrast maps to the group T1 template. Therefore, we first normalized each participant's mean functional EPI to the participant's structural T1 image and then to the T1 group template by using FSL “epi_reg” (Jenkinson and Smith, 2001; Jenkinson et al., 2002; Greve and Fischl, 2009) and ANTS “WarpImageMultiTransform.sh”, respectively (Avants et al., 2010, 2011). This procedure resulted in participant-specific transformation matrices that could then be used for the spatial normalization of the contrast maps.

##### Second-level group analyses.

For group analyses, we assessed consistent differences in functional activity across participants. Therefore, the spatially normalized contrast maps from each participant were entered into a general linear model using SPM12 (Penny et al., 2011). Unless stated otherwise, group results are reported with an initial cluster-defining threshold of *p* < 0.005.

#### Functional analyses in detail

Two participants were excluded from all functional analyses due to an amount of outlier scans exceeding 10% of the total scans at retrieval. Outliers were determined by excessive motion (threshold 2 mm) or global signal changes (threshold 9.0). In addition, all ROI analyses within hippocampal subfields were conducted with one participant less because of motion in the T2 image of that participant, which made hippocampal subfield segmentation impossible.

For all analyses the object and animal conditions were merged (Horner et al., 2015). Note, that we did not see any specific functional activity for animals in the “retrieval phase: element-specific activity” analysis (see below). When lowering the threshold (*p* < 0.005, uncorrected), however, functional clusters were comparable to the object condition (in lateral occipital cortex). As we did not see differences in functional activity, we collapsed object and animal conditions to assure comparability of results with Horner et al. (2015). The animal and the object condition will both be referred to as the object category in the following.

#### Retrieval phase: element-specific activity

To examine significant clusters of functional activity related to specific categories of event elements, we set up a general linear model with seven regressors-of-interest. Each regressor included the boxcar convolved stimulus onsets for one type of cue–target association (location–object; object–location; object–people; people–object; people–location; location–people). Each trial duration was determined by the response time. An additional regressor was included that modeled the interstimulus interval with a duration of 1.5 s. To assess differences in functional activity related to the three element categories, contrast maps were obtained between the parameter estimates related to the regressors that contained the respective category and those that did not contain the respective category. For instance, to obtain location related clusters of significant functional activity, we contrasted the parameter estimates obtained for the location–object, object–location, location–people, and people–location regressors with the parameter estimates for the object–people and people–object regressors.

To examine consistent clusters of significant functional activity at group level, the normalized contrast maps were entered into a one sample *t* test on the second level. All results are reported with familywise error correction after applying an initial cluster-defining threshold of *p* < 0.001.

#### Cortical reinstatement at retrieval

Here, we initially evaluated whether the function an element occupies at retrieval (cue, target, or nontarget) entails differences in the overall amount of cortical reinstatement. Subsequently, differences in cortical reinstatement of cues, targets, and nontargets between closed- and open-loop events were explored.

**Figure 3.**
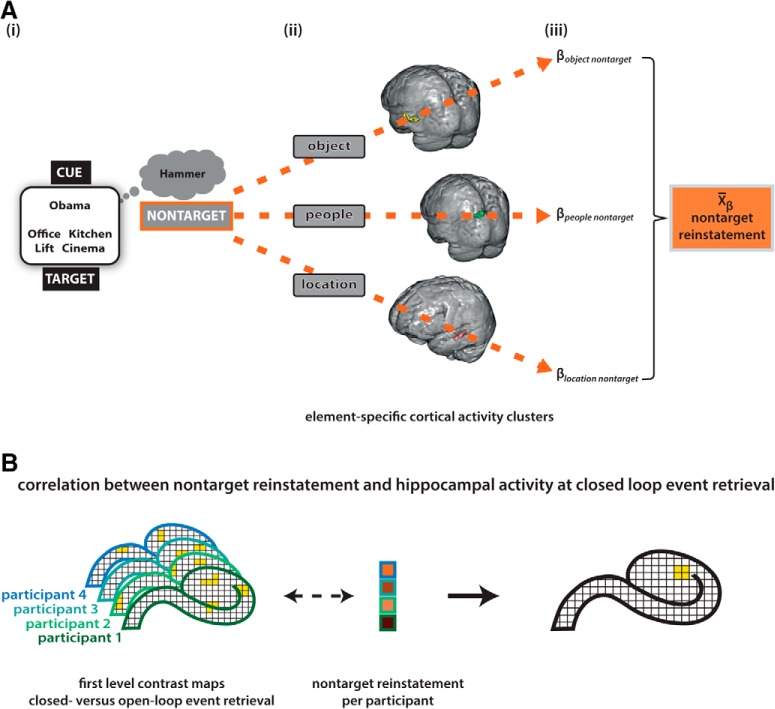
Overview “hippocampal activity–nontarget reinstatement” analysis procedure. ***A***, Calculation of participant-specific nontarget reinstatement values. At each retrieval trial one event element serves as a cue and one is the target. The additional element remains incidental to the task; that is the nontarget (***Ai***). From the previous “element-specific activity at retrieval” analysis, cortical clusters have been identified that specifically relate to the respective element categories (i.e., PHC for location, MPC for people, LOC for object; ***Aii***). For each participant, β values are extracted from the respective cluster for the condition that the category's function at retrieval is to be a nontarget (***Aiii***). *Z*-standardized β values are averaged subsequently to obtain an overall nontarget reinstatement value per participant. ***B***, Correlations between nontarget cortical reinstatement and hippocampal activity. With a univariate first level GLM analysis, participant-specific contrast maps are obtained that indicate the difference in hippocampal activity between the closed- and open-loop retrieval condition. At the group level that hippocampal activity pattern was correlated with the participant-specific nontarget reinstatement values. This yielded a statistical map, indicating hippocampal activity at closed-loop retrieval that was scaled by the amount of nontarget reinstatement across participants. PHC, parahippocampal cortex; MPC, medial parietal cortex; LOC, lateral occipital cortex.

To begin with, the amount of cortical reinstatement was assessed for each function an element could take (cue, target, and nontarget), across event loop conditions. This yielded an overall cortical reinstatement score per element function and participant (Fig. 3*A*). Based on the previous analysis (retrieval phase, element-specific activity) we obtained a significant cortical functional cluster for each category (location, people, and object) at the group level (Fig. 3*Aii*). In the case of multiple significant functional clusters, we focused on the element-specific ROI that was identified by Horner et al. (2015) to assure comparability of results (note that we obtained comparable results when using all our identified clusters). The corresponding functional masks were coregistered to each participant's native space with FSL FLIRT (Jenkinson and Smith, 2001; Jenkinson et al., 2002; Greve and Fischl, 2009). Using REX (RRID:SCR_005994; Whitfield-Gabrieli, 2009), we then extracted participant-specific parameter estimates for each regressor of interest in the element-specific activity analysis out of each element-specific ROI. Parameter estimates within each ROI were *z*-standardized. To obtain a participant-specific value for the amount of cortical reinstatement related to each element function, we took the parameter estimates out of each ROI, first for the condition that the respective ROI was related to the category of the cue (“cue cortical reinstatement”), second for the condition that the respective ROI was related to the category of the target (“target cortical reinstatement”), and third for the condition that the respective ROI was neither related to the category of the cue or the target but only related to the nontarget category (“nontarget cortical reinstatement”; Fig. 3*A*). For instance, the previous analysis (element-specific activity at retrieval) found a significant cluster of increased functional activity in the parahippocampal cortex for location category stimuli. Now, we took the parameter estimate regarding the people–object and object–people condition out of the parahippocampal cortex to obtain a measure for the nontarget cortical reinstatement for when the location was nontarget. Similarly we proceeded for the remaining two categories (people, object) to obtain nontarget cortical reinstatement values for each category. The normalized parameter estimates were averaged across ROIs (i.e., categories) for each participant, separately for cue, target and nontarget cortical reinstatement (Fig. 3*Aiii*). Differences in the amount of overall cortical reinstatement between element functions (cue, target, nontarget) were tested using a repeated-measures ANOVA.

To further explore the differences in cortical reinstatement between closed- and open-loop events, we then evaluated cortical functional activity for both event loop conditions. To compare cortical reinstatement between event loop conditions, we had to delineate functional cortical activity for closed- and open-loop events. Therefore, the above described univariate analysis (element-specific activity at retrieval) was performed again. Instead of 7 regressors of interest, 14 were created, they contained the same information as the 7 in the analysis before, now split up into trials that belonged to closed-loop and open-loop events. Then, the same procedure was followed as described in the previous paragraph to acquire element-related cortical activity values for cue, target, and nontargets per participant. Now however, calculated for closed-loop events and open-loop events separately. Subsequently, obtained difference scores for cortical reinstatement between event loop conditions were tested for significant deviation from zero by using one-sample *t* tests to assess whether cortical reinstatement was higher in closed-loop events.

#### Hippocampal activity and cortical reinstatement

The following analyses were aimed to identify activity clusters in the hippocampus that functionally relate to holistic recollection and to delineate their subfield-specific localization. As holistic recollection is conceptualized to be measurable by the amount of nontarget cortical reinstatement, we assessed hippocampal functional correlates of increased nontarget cortical reinstatement in closed-loop events.

We first followed an exploratory parametric analysis approach to assess whether any hippocampal cluster correlates with nontarget cortical reinstatement under conditions of increased holistic recollection. Therefore, initially a univariate first-level analysis was performed. The general linear model encompassed three regressors-of-interest. One contained the boxcar function convolved stimulus onsets for trials that are part of closed-loop events (duration equaled response time). The second regressor contained the boxcar function convolved stimulus onsets for trials that belong to open-loop events (duration equaled response time). The third regressor contained the boxcar convolved onsets of the interstimulus intervals (duration 1.5 s). Contrast maps were obtained for each participant for closed-loop versus open-loop event retrieval trials.

To investigate hippocampal involvement in holistic recollection, that is particularly the cortical reinstatement of nontargets, we used the first-level contrast maps that indicated for each individual where in the hippocampus BOLD activity was greater for closed-loop than open-loop event retrieval (Fig. 3*B*). With the second-level group analysis, we investigated which of the functional activity clusters that related to closed-loop retrieval correlate with the amount of nontarget cortical reinstatement across participants (Fig. 3*B*). To assess the functional specificity of the revealed significant cluster at nontarget cortical reinstatement, the second level group analysis was performed two more times, additionally for cue cortical reinstatement and target cortical reinstatement. Each general linear model included the normalized contrast maps for the contrast closed > open-loop retrieval of each participant as a first regressor. The second regressor included the respective participant-specific value for cue, target, or nontarget reinstatement, obtained by the independent analysis of element-category related cortical activity at retrieval (Fig. 3*A*). All results are reported with an initial cluster-defining threshold of *p* < 0.005. Small volume correction with a bilateral hippocampal mask was applied at second level.

To assess whether the identified hippocampal cluster correlated more with nontarget cortical reinstatement than with cue or target reinstatement, participant-specific mean functional activity was extracted from the respective cluster for the contrast closed > open-loop retrieval with REX (Whitfield-Gabrieli, 2009). Pearson correlation coefficients for each cortical reinstatement type (cue, target, and nontarget) with the extracted functional cluster activity were obtained. With a one-tailed *z* test we tested whether the obtained Pearson correlation coefficients were significantly higher for nontarget reinstatement than for cue and target reinstatement respectively (Rosenthal et al., 1992; Diedenhofen and Musch, 2015).

The clusters identified by above described analyses can only be attributed to a specific subfield by visual inspection. As they were considered to be located close to the right anterior CA3–DG border, a subsequent ROI analysis was performed to delineate functional involvement of CA3 versus DG. Therefore, mean β values from the first level analyses were extracted using REX (Whitfield-Gabrieli, 2009) for each individual out of the manually segmented hippocampal subfields masks for right anterior CA3 and right anterior DG. Beta values were extracted referring to the closed-loop regressor and to the open-loop regressor. Pearson correlation coefficients and corresponding significance values were obtained for the relationship between the difference in β values (closed- vs open-loop) and the amount of nontarget reinstatement across participants. With a one-tailed *z* test we tested whether the obtained Pearson correlation coefficient was significantly higher for right anterior CA3 than right anterior DG (Rosenthal et al., 1992; Diedenhofen and Musch, 2015).

## Results

### Behavioral results

On average 87.41% (SD = 9.78%) of all trials in the recall phase were answered correctly by the 30 participants. There was no significant difference in accuracy between closed-loop (mean = 88.55%, SD = 8.96%) and open-loop events (mean = 86.27%, SD = 10.60%).

We also investigated the amount of dependency among event elements. Note, that the dependency measure we calculated scales with accuracy. Therefore the evidence for dependency is defined as the difference between data-based dependency and the expected dependency based on the independent model. The evidence for dependency is not significantly higher for closed- than open-loop events (*t*_(29)_ = 1.162; *p* = 0.255). The higher the overall accuracy, the more dependency values approach 1 (Horner et al., 2015). Our very high accuracy may thus have led to ceiling levels in the estimated dependency measures, making it impossible to detect differences between open- and closed-loop event dependency.

**Figure 4.**
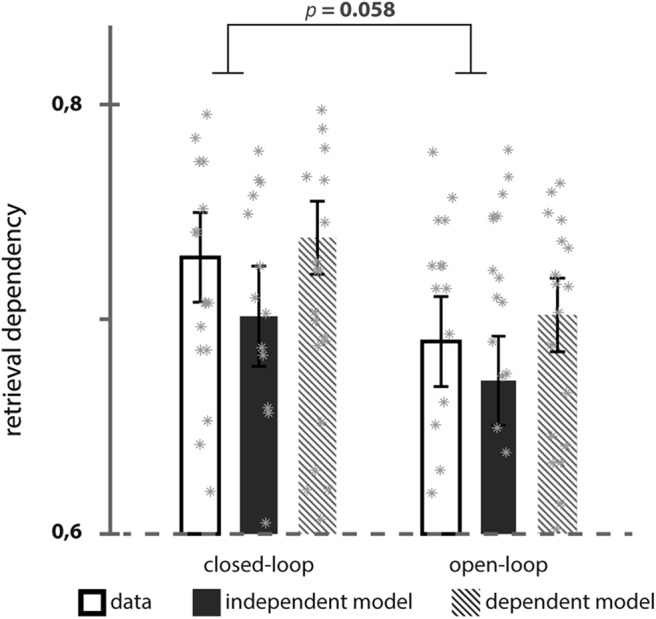
Behavioral dependency between multiple retrieval trials from closed- and open-loop events. Observed dependency between trials from the same event was compared with estimated dependency assuming fully independent and dependent models. Note that here depicted dependency is calculated based on high confidence (Levels 3–4) versus collapsed low confidence (Levels 1–2) and incorrect retrieval trials. Error bars ±1 SE.

To test whether the high overall accuracy may have obscured stronger dependency among closed-loop elements, we calculated dependency again by taking the confidence level into account. That is, instead of classifying the retrieval trials by correct versus incorrect, we split them into high and low confidence trials and collapsed incorrect and low confidence trials. The evidence for dependency is not significantly different between loop conditions (*t*_(29)_ = 1.978; *p* = 0.058). However, open-loop events but not closed-loop events showed significantly lower dependency than the dependent model (*t*_(29)_ = −2.59; *p* = 0.015; *t*_(29)_ = −1.47; *p* = 0.152). Numerically, our results are consistent with previous results (Horner et al., 2014, 2015). That is, retrieval at closed-loop events entails more dependency among event elements than retrieval at open-loop events (Fig. 4).

### Univariate results

#### Element-specific cortical activity at retrieval

The aim of this analysis was to identify element-specific cortical functional activity patterns at retrieval. Therefore, category associations that contained a respective element were contrasted with category associations that did not contain the respective element (e.g., identify location activity by contrasting location–object and location–people with people–object trials).

People-related activity was found in the medial parietal lobe (cluster size: *k* = 1172, *p* < 0.001; Fig. 3*Ai*), in a left inferior temporal cluster (cluster size: *k* = 103, *p* = 0.006) and in a right lateral parietal cluster (cluster size: *k* = 126, *p* = 0.001). Object-related activity was found in the left lateral occipital lobe (separated into three clusters: first cluster size: *k* = 864, *p* < 0.001; Fig. 3*Ai*; second cluster size: *k* = 101, *p* = 0.006; third cluster size: *k* = 75, *p* = 0.041). Location-related activity was found in bilateral clusters in the parahippocampal cortex (left cluster size: *k* = 2242, *p* < 0.001, right cluster size: *k* = 883, *p* < 0.001; Fig. 3*Ai*), bilateral retrosplenial cortex (cluster size: *k* = 7786, *p* < 0.001) and bilateral lateral parietal cortex (left cluster size: *k* = 698, *p* < 0.001, right cluster: size *k* = 418, *p* < 0.001).

#### Cortical reinstatement during closed-loop event retrieval

The identification of element-specific activity patterns at retrieval allowed us to obtain participant-specific values for the amount of cortical reinstatement at retrieval (Fig. 3*A*). Therefore, parameter estimates were extracted from each element-specific cortical region when the respective element functioned as a cue, target, or nontarget. We averaged these values across element categories. Note that, when multiple element-specific clusters have been identified, we extracted parameter estimates exclusively from the region selected by Horner et al. (2015) to assure comparability of results (i.e., people: medial parietal cluster, animal/object: left lateral occipital cluster, location: bilateral parahippocampal cluster). Thus, we obtained three values per participant that reflect the element-related cortical activity at retrieval: first, the cue cortical reinstatement, thus the functional cortical activity induced by cues; second, the target cortical reinstatement, that is functional cortical activity induced by targets; and third, the cortical reinstatement of nontargets, i.e., the cortical reinstatement of event elements currently incidental to the task.

**Figure 5.**
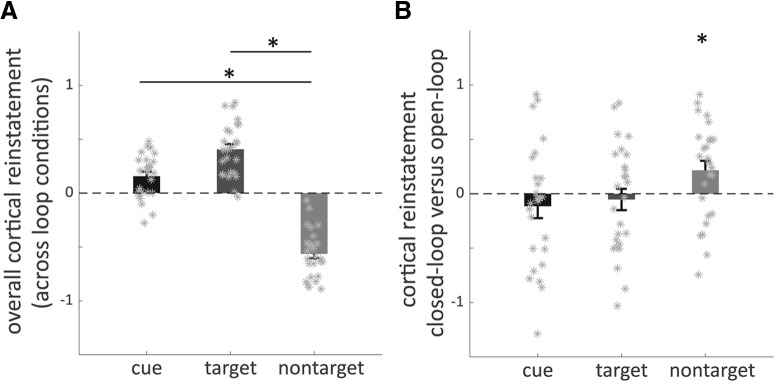
Difference in cortical reinstatement between element functions (i.e., cue, target, nontarget; ***A***) across loop conditions (“overall” cortical reinstatement) and (***B***) subtracting cortical reinstatement at open-loop from closed-loop retrieval. ***A***, *Denotes significant difference (*p* < 0.05). ***B***, *Denotes significant difference from zero (*p* < 0.05).

Over all experimental conditions, cue and target cortical reinstatement was significantly higher than nontarget cortical reinstatement, and targets induced significantly more cortical activity than cue elements (Fig. 5*A*; main effect of element function: *F*_(2,75)_ = 111.35; *p* < 0.001, ANOVA). Note that the displayed β values are not in relationship to an explicit baseline but rather the overall mean parameter estimate. Differences are thus not absolute but relative to each other. We operationalized holistic recollection as the amount of incidental reinstatement, i.e., reactivation corresponding to nontarget elements. To test whether closed-loop event retrieval entails more holistic recollection, we investigated whether more nontarget cortical reinstatement took place for closed-loop than open-loop event retrieval (Fig. 3*B*). Indeed, the difference between the amount of element-related cortical activity in closed- and open-loop conditions is only significantly higher than zero for nontargets (*t*_(25)_ = 2.46, *p* = 0.02), not so for cues (*t*_(25)_ = −1.04, *p* > 0.05) or targets (*t*_(25)_ = −0.05, *p* > 0.05; Fig. 5*B*; one-sample *t* tests). Thus, cortical reinstatement of nontargets was higher for closed-loop than open-loop retrieval.

#### Anterior CA3, but not DG activity during closed-loop retrieval correlates with overall nontarget reinstatement

Phenomenological differences between closed- and open-loop retrieval in terms of holistic recollection, i.e., the amount of nontarget cortical reinstatement, are apparent based on the previous analyses. We therefore examined whether there are specific hippocampal functional correlates of closed-loop event retrieval. When functional differences between closed- and open-loop event retrieval are related to holistic recollection, they should scale with the amount of nontarget reinstatement a participant engages in.

**Figure 6.**
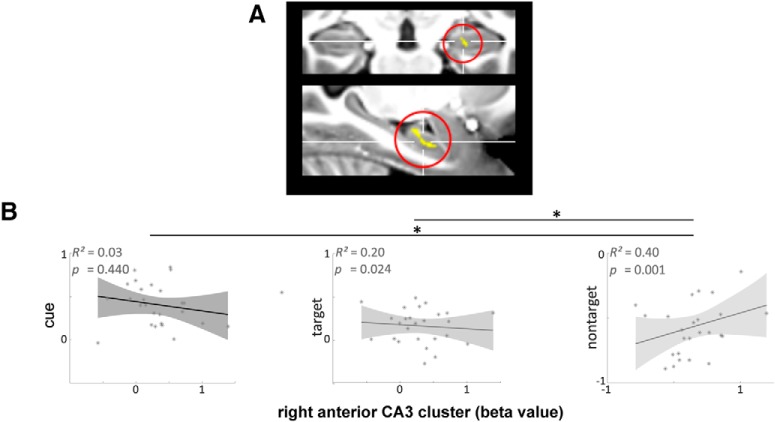
Functional hippocampal activity correlations at closed-loop retrieval with overall nontarget cortical reinstatement. ***A***, Hippocampal cluster whose difference in activity between retrieval of closed- versus open-loop events correlates with amount of nontarget reinstatement across participants [cluster size *k* = 35; *p*(cluster) = 0.028 (uncorr)]. ***B***, Correlations between cue, target, and nontarget cortical reinstatement, and the extracted β values for closed- versus open-loop retrievals from the identified hippocampal cluster, respectively. *Denotes significant differences between correlations (*p* < 0.05).

First, we contrasted BOLD activity during closed- and open-loop event retrieval within each participant. This yielded participant-specific statistical maps indicating functional activity differences between both loop structures. At the group level these contrast maps were then correlated with the participant-specific amount of nontarget cortical reinstatement. This explorative approach yields clusters within the hippocampus that display increased functional involvement during closed-loop event retrieval when overall nontarget cortical reinstatement, i.e., holistic recollection, is high (Fig. 3*B*). An anterior right hippocampal cluster [cluster size *k* = 35; *p*(cluster) = 0.028 (uncorr)], located in subfield CA3, was revealed that scales its functional activity during closed-loop event retrieval with the participant's amount of overall nontarget cortical reinstatement (Fig. 6*A*). Note, that no significant clusters could be identified for the reverse correlation and when correlating individual contrast maps for open > closed-loop retrieval with the overall nontarget cortical reinstatement across individuals.

To test whether the identified cluster was specific for nontarget reinstatement, i.e., holistic recollection, and not related to other retrieval processes, we first tested whether the respective cluster correlated with cue and target reinstatement as well. Pearson correlations between cluster activity (i.e., extracted β values for the closed–open-loop contrast) and cue as well as target reinstatement were significantly lower than the previously identified correlation of the right anterior CA3 cluster with nontarget reinstatement [*z* = −2.584, *p* = 0.005 and *z* = −3.226, *p* = 0.001 for the difference in correlations between *p*(nontarget reinstatement, cluster activity) and *p*(cue reinstatement, cluster activity) or *p*(target reinstatement, cluster activity), respectively]. Second, we investigated whether additional anterior hippocampal activity is related to cue- or target-induced cortical activity. Therefore, the same parametric analyses approach was adopted at group level as we applied for the identification of hippocampal activity related to nontarget reinstatement. Now, however we correlated the difference in functional activity between loop conditions with cue and target cortical reinstatement respectively. No anterior hippocampal cluster showed increased involvement during closed-loop event retrieval with higher amounts of cue or target cortical reinstatement. Together, we identified a cluster, located in anterior right hippocampal subfield CA3, where activity during closed-loop retrieval correlates with the amount of overall nontarget cortical reinstatement in each participant.

**Figure 7.**
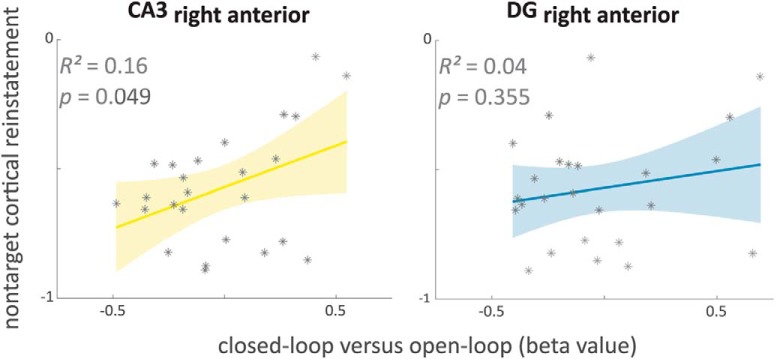
Functional activity correlations of subfield ROIs at closed-loop retrieval with overall nontarget cortical reinstatement. Differences in activity between closed- and open-loop retrieval were extracted as mean values from manually segmented hippocampal subfields CA3 and DG (right anterior) and subsequently correlated with the amount of overall nontarget cortical reinstatement. CA3, cornu ammonis 3; DG, dentate gyrus.

So far, only by visual inspection we assigned the identified right anterior hippocampal cluster to subfield CA3. As the cluster is in close vicinity to the DG, we aimed to disentangle the specific contributions. Therefore, a ROI approach was adopted. We extracted functional activity (β values) from manually segmented right anterior subfield CA3 and DG, respectively, for the loop condition contrast (closed- > open-loop event retrieval). The mean functional activity within ROIs was correlated with the amount of nontarget cortical reinstatement across participants. Indeed, only for the right anterior CA3 but not for the right anterior DG the mean functional activity was correlated with the overall amount of nontarget cortical reinstatement across participants (Fig. 7; *R*^2^ = 0.16, *p* = 0.049; *R*^2^ = 0.04, *p* = 0.355 for the correlation nontarget cortical reinstatement, right anterior CA3 and DG, respectively). The correlation between nontarget cortical reinstatement and right anterior CA3 was, however, not significantly higher than with right anterior DG (*z* = 1.088, *p* = 0.138). The ROI results are further evidence for a trend toward specific functional involvement of subfield CA3 (right anterior) but less of adjacent subfield DG in closed-loop event retrieval when participants generally entail more nontarget cortical reinstatement.

## Discussion

Using ultra-high resolution 7 tesla fMRI, we provide first empirical evidence for the involvement of human hippocampal subfield CA3 in holistic recollection via pattern completion. Therein we go beyond a replication of the main findings by Horner et al. (2015) and unpack the functional involvement of hippocampal subfields at recollection of multi-element events.

Our paradigm relies upon the assumption that multi-element events composed as a closed-loop entail more holistic recollection at retrieval than events with an open-loop structure. Extensive previous research provides support for an increased dependency among event elements that are encoded in an all-to-all associative manner (Horner and Burgess, 2013, 2014; Horner et al., 2015). The likelihood to incidentally retrieve event elements when cued with one element, i.e., for holistic recollection is therefore increased in closed-loop events. Consequently, cortical reinstatement of incidental event elements has been shown and here again been confirmed to be higher when retrieving closed-loop events (Horner et al., 2015; Figs. 4, 5). We additionally demonstrated increased functional involvement of right anterior subfield CA3 at closed-loop event retrieval in relation to cortical reinstatement of incidental elements (Fig. 6*A*). Our data indicate that anterior CA3 activity is related to successful pattern completion associated with holistic recollection. Thereby we contribute to recent efforts in empirically addressing the functional subfield architecture of the human hippocampus.

Although models of the functional organization of hippocampal subfields (Amaral and Witter, 1989; Lisman, 1999; Hunsaker and Kesner, 2013) have been informed by anatomical and animal research, the translation of these insights to humans has been limited by the resolution of fMRI, particularly in distinguishing functional activity in CA3 and DG. Here, we were able to acquire functional images with a submillimeter resolution (0.8 mm isotropic) allowing us to segment CA3 and DG separately and to examine specific functional patterns of both subfields (Berron et al., 2016). Indeed, the anatomical ROI analysis confirms that the association between functional subfield activity and the amount of holistic recollection particularly holds for anterior CA3 but less for the adjacent DG (Fig. 7). The association between subfield CA3 and a condition that entails more pattern completion is in accordance with previous animal research (Nakazawa et al., 2002; Lee and Kesner, 2004; Vazdarjanova and Guzowski, 2004; Gold and Kesner, 2005; Fellini et al., 2009; Neunuebel and Knierim, 2014).

Despite proposed anatomical and functional heterogeneity between hippocampal subfields, recent human functional imaging showed functional heterogeneity along the longitudinal axis (Collin et al., 2015; Brunec et al., 2018). Interestingly, proposals exist for scene imagination, transitive inference processes, and pattern completion being related to the anterior hippocampus (Poppenk et al., 2013; Strange et al., 2014; Zeidman and Maguire, 2016). Our finding of anterior hippocampal involvement in holistic recollection might be seen in line with that literature.

Also along the transversal axis of the hippocampus considerable heterogeneity has been suggested. Importantly, the anatomical transition between subfields is not decisive but rather graded (Amaral and Witter, 1989). This renders it difficult to strictly examine functional activity of CA3 and DG independently. Moreover, despite the usage of ultra-high resolution functional imaging, 2 mm smoothing was applied, which blurs functional data at the border of segmented subfields. Nevertheless, our anatomical ROI analysis averages functional signal across whole subfields that extend more than the 2 mm smoothing radius. The observed significant correlation between CA3 activity and holistic recollection is thus, even though not completely independent from DG activity, a confirmation of CA3 being significantly involved at successful holistic recollection.

Particularly in the anterior medial part (i.e., uncal region), hippocampal anatomy is highly complex and variable between individuals (Ding and Van Hoesen, 2015). Therefore, some subfield segmentation protocols decided to spare this region (Dalton et al., 2017). Indeed, subfield-specific interpretations in the hippocampal head should be drawn with caution. However, the segmentation protocol, that we have applied, leveraged the higher resolution at 7T (i.e., 1 mm slice thickness) to translate recent findings on subfield boundaries in the hippocampal head from neuroanatomy to MRI (Ding and Van Hoesen, 2015; Berron et al., 2017).

Note, that the cortical reinstatement of incidental elements (“nontargets”; Fig. 3) is an indirect measure for hippocampal pattern completion. Theoretical models propose that successful retrieval is initiated by completing a cue pattern toward the full event representation in the hippocampus (Marr, 1971; Treves and Rolls, 1994; McClelland et al., 1995). Pattern completion may go beyond the required target and include nontargets, particularly if the event representation binds multiple elements tightly together (Horner and Burgess, 2014; as, e.g., in closed-loop events; Horner et al., 2015). The elements of the completed event representation are subsequently reinstated in the cortex, which then creates a recollective experience (Staresina et al., 2012, 2013; Bosch et al., 2014; Gordon et al., 2014; Thakral et al., 2015; Liang and Preston, 2017). Thus, our observation of increased cortical activity associated with incidental event elements upon retrieval, and its correlation with activity in CA3 supports these models and implicates CA3 in hippocampal pattern completion and holistic recollection.

Even though our measure of pattern completion is indirect, several aspects of our results support the specific involvement of anterior CA3 in holistic recollection. First, the anterior CA3 cluster related to cortical reinstatement of nontargets could not be identified in relationship to cue or target cortical activity and functional activity within the CA3 cluster was not correlated with reinstatement of cues or targets (Fig. 6*B*). Because cues and targets are presented on screen, successful pattern completion is less relevant for the retrieval of these elements. The increased activity of anterior CA3 at closed-loop event recollection when nontarget cortical reinstatement is high, can thus be referred back to the increased engagement of a pattern completion mechanism (Horner et al., 2015). Second, the anterior CA3 involvement at closed-loop event retrieval cannot be explained by mere recall success. Despite more holistic recollection at closed-loop events (i.e., higher retrieval dependency and more nontarget reinstatement), accuracy levels in both event structure conditions are similar. This rules out performance to be a driving factor in the functional activity pattern of anterior CA3. Importantly, we observed CA3 activity in relation to the amount of holistic recollection during the whole task, averaged across both event loop conditions (i.e., in relation to overall holistic recollection). Thus, participants that generally engaged in more holistic recollection, showed more CA3 activity when retrieving closed-loop events. In contrast, Horner et al. (2015) observed that hippocampal involvement at retrieval of closed-loop events increased with the difference in holistic recollection between closed- and open-loop events. Small variations in our data may explain the subtle differences in results. Even though we similarly observed higher nontarget reinstatement at retrieval of closed-loop events (Fig. 5), the difference to nontarget reinstatement at open-loop events was smaller than that shown by Horner et al. (2015). In our data, performance in both loop conditions was higher and there was more holistic recollection in open-loop events (Fig. 4; perhaps due to higher performing participants inferring the missing associations), so that differences between closed- and open-loop events were reduced.

Although we leveraged the closed- versus open-loop contrast to examine specific hippocampal involvement during holistic recollection via pattern completion, we do not claim that the hippocampus is not involved in the recollection of open-loop associations. The hippocampus likely mediates the associative memory required to answer the paired-associate questions regarding both open- and closed-loop events. However, the open-loop events serve as a strict control condition, as our data and previous literature indicate that there will be greater pattern completion for closed-loop events, resulting in tighter dependency among elements and greater incidental reactivation of nontarget elements (Horner and Burgess, 2014; Horner et al., 2015). Pattern completion is defined as a computational mechanism on the representational level (Treves and Rolls, 1994; McClelland et al., 1995). We, however, took a univariate analysis approach here. Moreover, as we averaged across trials and restricted our cortical reinstatement analysis to ROIs, we may not have captured the full variety in the functional activity pattern at holistic recollection. Future studies need to verify pattern completion mechanisms in the human CA3 on trial-specific level as well as directly on representational level by multivariate approaches. The hippocampal effects need to be related to cortical reinstatement beyond our restricted ROIs. In addition, future ultra-high resolution neuroimaging studies should dissect the potential heterogeneity in the functional architecture along the hippocampal axes. Such spatially and temporally more fine-grained analyses will have the potential to show pattern completion effects in the human brain more explicitly.

To sum up, we acquired functional data in ultra-high resolution with 7 tesla fMRI using the established multi-element event paradigm by Horner et al. (2015). In accordance with anatomical and animal research, our results yield the first compelling empirical evidence for a functional involvement of the human hippocampal subfield CA3 (but less pronounced in DG) in holistic recollection via pattern completion. The current study contributes to our understanding of the heterogeneous functional architecture within the human hippocampus.
